# Puerperal Infection

**Published:** 1902-01

**Authors:** W. Monroe Smith

**Affiliations:** Atlanta, Ga.


### PUERPERAL INFECTION.*

BY W. MONROE SMITH, M. D.,

ATLANTA, GA.

I feel some timidity in selecting as a topic for my remarks before this esteemed society, to-night a subject which has been recognized and discussed for twenty centuries and more; but offer as an apology for so doing, the fact that this is a disease which still exists with as much virulence, sacrificing the lives of some mothers and threatening the lives of many more.

It is a condition not infrequently met by every physician who attends the puerperal woman, and is dreaded by all on account of the uncertainty of the results.

It is always caused from infection conveyed from without and never originates from within the body of the patient; consequently it must be a preventable disease. In the time of Hippocrates, Galen and Ambrose Paré, it was thought to be due to suppression of the lochia, an impression which still holds sway to a large extent in the minds of the laity to-day.

*Read before Atlanta Society of Medicine, Oct. 1901.

Various micrococci have been isolated as causative factors of this condition, and the same cocci produce different local lesions and to a less extent different constitutional symptoms in different cases. The streptococci are the kind most generally found and produce the most pronounced lesions.

In one case infection will only invade the endometrium; in other cases the body of the uterus, the tubes, the cellular tissue and peritoneum are involved as well. The heart, the lungs, the plura and kidneys are often invaded during the progress of this disease,—in fact there seems to be no organ of the body which is immune from an attack. The time of development is usually about the third day after labor, but is often delayed to the fifth or sixth day and in some cases ten to twelve days, or even longer. The first symptoms are usually that of a chill, fever, headache and general aching pains over the entire body, with or without localized pain and tenderness.

If inflammation be present there will, of course, be localized pain and tenderness corresponding to the organ or organs involved. There may be only one chill followed by continued fever or there may be repeated chills followed by intermission or remission in the fever. Repeated chills and fever, followed by intermissions in the temperature without local lesions, are due to decomposing coagula or portions of retained ovum, the symptoms of which quickly subside upon the complete emptying of the uterus.

While as a rule the symptoms are thus well defined, yet in many cases there will be no chill, but fever, pain, tenderness and general bad feelings, etc.

The condition is to be differentiated from typhoid fever, malarial fever, diarrhea, etc., and while usually not difficult, yet to some physicians it is not always an easy task.

There is something in the general aspect of a patient suffering from septic infection, which aids greatly in distinguishing it from other conditions with similar symptoms.

The pulse is quicker than would be the case in other conditions with same amount of fever, the expression is more anxious and the patient has the appearance of being sicker than in other diseases, which might be mistaken for it.

Then in most cases you have localized symptoms, foul lochia, etc., which are pathognomonic of this disease.

But recently I had a case under my own observation which developed the first symptoms of infection on the fourteenth day after labor and was characterized by chill, fever, headache and sweat, then intermission of symptoms to be followed forty-eight hours later by another chill, fever, etc. When I was called to see her, had it not been for that condition which I recognized by sight, indicating sepsis, owing to the long interval from labor to symptoms of the infection, I should have diagnosed malaria, but as a doubt arose, I hunted the blood carefully for plasmodium malaria, and finding none, I curetted my patient's womb and douched it out carefully and was rewarded by a rapid recovery, without having to use quinine.

Another case where labor was followed on the second day by chill, high fever, headache, etc., and sweat, yet without that appearance of being seriously ill, blood examination showed the malarial infection and recovery promptly followed antimalarial treatment.

In some cases there are symptoms of mild infection, but these will generally be distinguished by local symptoms of suflumation, well walled off and consequently devoid of the severer constitutional symptoms , which characterize the more general infections.

As to treatment the most that can be done is preventative measures. The prospective mother should be kept in as good general health as possible, the general functions should be carefully watched and any diseased condition corrected, especially is this true as to the kidneys. The confinement bed should be prepared as well as the patient with clean linen ; the practice among many old women of preserving old worn-out quilts in some dirty cellar for the bed, of retaining on the body of the patient dirty gowns, for fear of soiling clean ones, etc., should never be countenanced, but the opposite enforced. The patient's genitalia should be carefully cleansed with soap and water and bichloride solution, and in delayed labors repeatedly sponged with bichloride solution.

The doctor's arms and hands must be carefully scrubbed with soap, water and brush, finger nails cleaned and cut short and hands soaked in bichloride solution and then rigidly kept sterile till labor is ended. After the labor is over, tears of the perineum should be carefully looked for and properly repaired and an antiseptic, or at least an asceptic dressing applied to the vulva and continued; and local antiseptic washes to the vulva used when change of dressing is necessitated.

Patient should be raised upright in bed several times daily for the passage of urine, which affords drainage of blood clots., etc. The fundus of the uterus should be gently kneaded daily to encourage uterine retraction and involution. I usually instruct my patients how to do this latter and they generally find amusement in obeying this instruction.

But when symptoms of infection present, assure yourself that the uterus is empty by anasthetising patient and thoroughly curetting the uterus with the hand, the dull or sharp currette, or all as required, and thoroughly drenching the uterus with salt solution. Never use bichloride solution in an intra-uterine douche, especially during the curettage, or you may have cause to regret the fact that you did not heed this warning.

There is usually no necessity of packing the uterus after curettage, unless you have hemorrhage, but this will frequently happen when the uterus should be thoroughly packed with iodoform gauze. In exceptionally relaxed uteri without much hemorrhage, I sometimes pack to encourage better retractions.

After the uterus is once thoroughly emptied and cleansed, leave off intro-uterine douchings, as they will only stimulate trouble; use only vaginal antiseptic douches, apply ice bag over any localized inflammation, purge with salines in most cases freely and treat on general principles. Most cases require big liquid food, strychnine and whisky, etc.

If abscesses develop, they must be drained wherever located. The location of abscess will indicate the best plan of operation.

Pelvic abscesses should generally be drained through vagina.

With this plan of treatment but few patients should fail to recover. The most desperate cases usually recover.

Recently I had a case where I found a softened uterine wall, which could be easily invaded with finger during the curettage, general peritonitis, high fever, etc., where under the treatment outlined above the patient recovered sufficiently to be up and about, when symptoms of further sepsis were discovered, and upon examination to find the source an empyema was found, part of rib resected and patient finally made complete recovery.

In conclusion, I would advise thorough asepsis in management of labor cases, suspect all cases of diarrhoea attending confinement as septic in origin, and cleanse the uterus early upon the first positive symptom of infection, as it can be done safely then before the vital forces have been exhausted by sepsis, then treat symptoms on rational basis. Stimulate, but never depress your patient with antipyretics; use cold water for reduction of temperature. Treat your patients on advanced surgical principles and the great majority will recover.

